# Pretreatment with oral contraceptives benefit POSEIDON group 1 low prognosis patients during GnRH-antagonist protocol: a propensity score-matched retrospective cohort study

**DOI:** 10.1186/s13048-025-01613-6

**Published:** 2025-03-07

**Authors:** Ning Wu, Xin Li, Cheng Zeng, Jing Shang, Xiuli Yang, Qing Xue

**Affiliations:** Department of Obstetrics and Gynecology, Peking University First Hospital, Beijing, 134 P. R. China

**Keywords:** Oral contraceptive, Poor ovarian response, POSEIDON criteria, Clinical outcomes, Pretreatment

## Abstract

**Background:**

Poor ovarian response (POR) is a challenging condition in assisted reproduction technology. Oral contraceptives (OCs) are commonly used to suppress gonadotropin hormone release in POR patients to synchronize the development of antral follicles before ovarian stimulation. Nevertheless, the question of whether such approach confers advantageous outcomes has elicited inconclusive results in previous studies. Therefore, the objective of this study was to investigate the effect of OCs pretreatment in low prognosis patients stratified by Patient-Oriented Strategies Encompassing Individualized Oocyte Number (POSEIDON) criteria.

**Methods:**

This retrospective cohort study included 2,222 patients undergoing their first IVF or ICSI cycle from January 2012 to April 2022. After propensity score matching, 369 patients were in the OC pretreatment group and 879 in the control group. Patients were divided into four subgroups based on the POSEIDON criteria. Comparisons of ovarian response and clinical outcomes were conducted, and multivariable logistic regression was used to assess the association between OCs pretreatment and live birth, clinical pregnancy, and pregnancy loss rates.

**Results:**

Patients in POSEIDON group 1 who received OCs pretreatment exhibited a significant reduction in the dose and duration of gonadotropin administration, along with an increase in the number of oocytes retrieved, 2 pronuclei, available embryos, and good quality embryos, indicating an improvement in their ovarian response to exogenous gonadotropins. Additionally, the live birth rate (*P* = 0.030) and clinical pregnancy rate (*P* = 0.012) were significantly higher in the OCs pretreatment group. Multivariate logistic regression analysis demonstrated a positive association between OCs pretreatment and live birth rate (*P* = 0.008) and clinical pregnancy rate (*P* = 0.008). However, in POSEIDON group 2 to group 4, there were no significant differences in ovarian response or clinical outcomes between the OCs pretreatment group and the control group.

**Conclusions:**

Administering OCs as pretreatment prior to ovarian stimulation using gonadotrophin releasing hormone antagonist protocol appears to be a more favorable approach than waiting for natural menses in low prognosis patients belonging to POSEIDON group 1.

**Supplementary Information:**

The online version contains supplementary material available at 10.1186/s13048-025-01613-6.

## Background

The poor ovarian response (POR) is one of the major challenges in assisted reproduction technologies (ART). POR is characterized by low number of oocytes after ovarian stimulation, accompanied by the high level of exogenous gonadotropins (Gn), high cycle cancellation rate and low available embryos for implantation, all of which make conceiving more difficult [1].

The prevalence of POR ranges from 6 to 35% worldwide [2]. The lack of uniformity criteria leads to the wide range of occurrence and makes it difficult to evaluate the effectiveness of interventions. The Bologna criteria was proposed by European Society of Human Reproduction and Embryology (ESHRE) in 2011 to set a clear definition of POR, while the criteria were questioned because of the heterogeneity among the POR patients [1, 3]. In 2016, a more precise classification of low prognosis patients called Patient-Oriented Strategies Encompassing Individualized Oocyte Number (POSEIDON) criteria were established, which includes 4 subgroups based on age, ovarian reserve tests (anti-Mullerian hormone (AMH) or antral follicle count (AFC)) and previous ovarian stimulation response [4]. The POSEIDON criteria stratify POR primarily by age and defines unexpected suboptimal response, allowing for individualized treatment strategies for different types of patients [5]. Multiple adjuvant therapies such as androgens, coenzyme Q10, growth hormone and recombinant luteinizing hormone (LH) supplementation have been proposed to improve clinical outcomes on the specific subpopulations according to the POSEIDON criteria [6, 7].

Oral contraceptives (OCs), consisting of estrogen and progestogen, are widely used to prevent pregnancy and treat gynecology diseases like polycystic ovarian syndrome (PCOS) and menorrhagia [8]. Pretreatment with OCs before the ovarian stimulation in in-vitro fertilization (IVF) or intracytoplasmic sperm injection (ICSI) cycle could reduce cyst formation, scheduling for ovarian stimulation priming and reduce the amount of Gn administration [8, 9]. OCs pretreatment may aid synchronization of follicular development and enhancing the FSH sensitivity of antral follicles by inhibiting the pituitary production of follicle stimulating hormone (FSH) and LH through negative feedback, thus improving the ovarian response [10, 11]. Previous research indicates that ovarian stimulation with OCs pretreatment may improve the pregnancy outcomes in patients with POR [11–13]. Nevertheless, some investigators have indicated that pretreatment with OCs result in comparable or even inferior IVF outcomes as compared to those not receiving pretreatment in POR patients or in other patients undergoing ART [14–16]. Due to the lack of a clear and logical category for POR patients in the previous studies, the conclusion was ambiguous. This present study aimed to evaluate the administration of OCs prior to ovarian stimulation for low prognosis patients defined by POSEIDON criteria, providing new insights into the clinical application of OCs for different POR subpopulations, notably those patients uncovered by Bologna criteria.

## Methods

### Study population

**Fig. 1:**
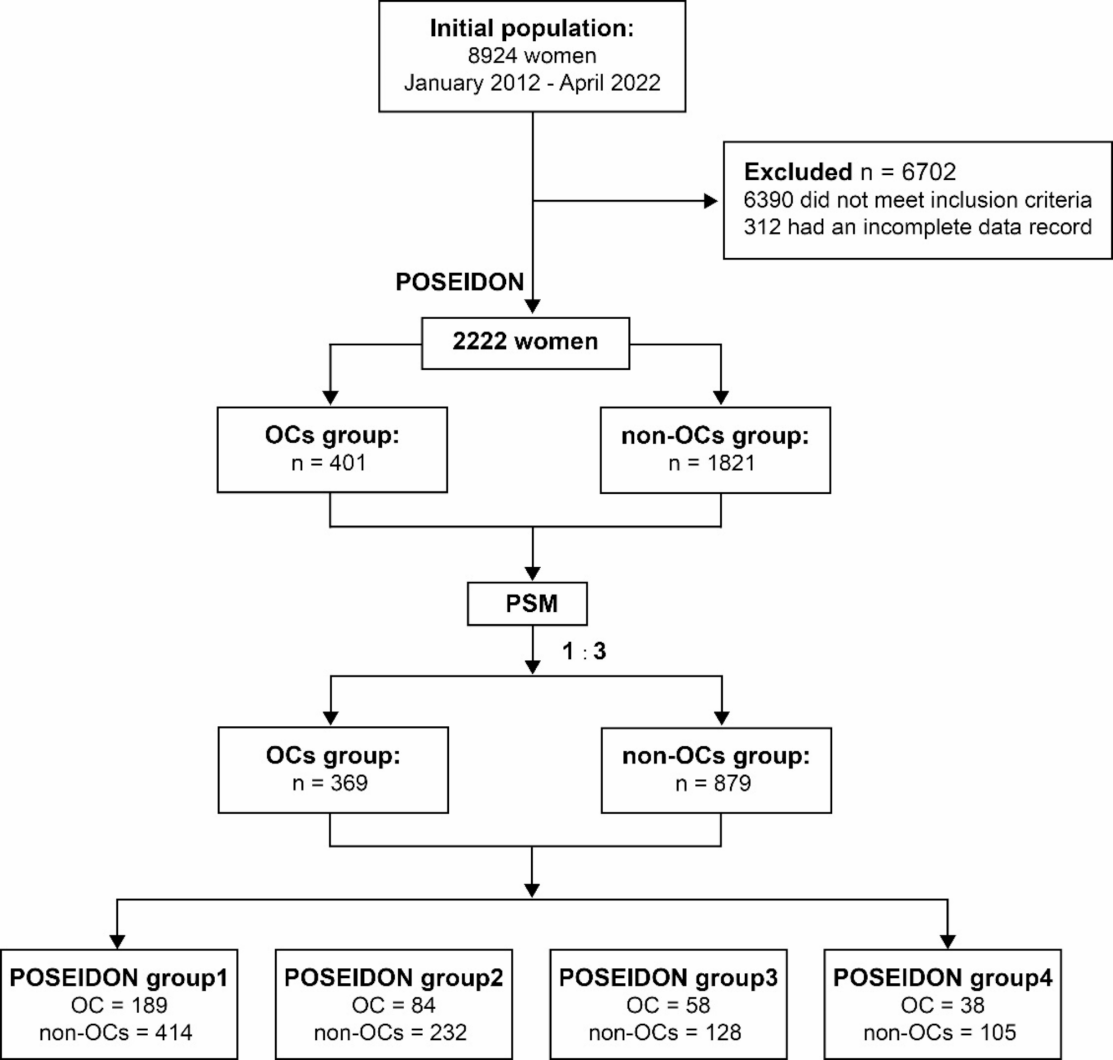
Flow chart of the study population

The present study conducted a retrospective cohort analysis of 2222 patients who were diagnosed with POR according to the POSEIDON criteria and underwent the first fresh IVF/ICSI cycles at the Reproductive Medicine Center of Peking University First Hospital between January 2012 to April 2022 (Fig. 1). The study was approved by the institutional ethics review board of Peking University First Hospital.

Due to the differences in age and ovarian reserve markers such as AFC and AMH between the OCs pretreatment group and the control group, a propensity sore matching (PSM) method was employed to rectify the uneven distribution of baseline characteristics at a ratio of 1:3 ratio with a caliper width equal to 0.01 [17]. This methodological approach aimed to minimize the potential confounding variables that may influence the analysis of patients who received OCs pretreatment and those in the control group.

After matching, the participants were classified into four subgroups based on the POSEIDON classification system [4]. Group 1 comprised patients < 35 years old with normal ovarian reserve parameters (AFC ≥ 5 and AMH ≥ 1.2 ng/mL) but with unexpectedly suboptimal ovarian response (number of oocytes retrieved < 10). Group 2 included patients ≥ 35 years old with normal ovarian reserve parameters (AFC ≥ 5 and AMH ≥ 1.2 ng/mL) but with unexpectedly suboptimal ovarian response (number of oocytes retrieved < 10). Group 3 consisted of patients < 35 years old with expected low ovarian response (AFC < 5 or AMH < 1.2 ng/mL). Group 4 included patients ≥ 35 years old with expected low ovarian response (AFC < 5 or AMH < 1.2 ng/mL).

The exclusion criteria were chromosomal abnormality, uterine malformations, polycystic ovarian syndrome, adenomyosis, leiomyoma (submucosal leiomyoma and fibroids ≥ 4 cm) and recurrent pregnancy loss.

Clinical and treatment-related data were obtained from the electronic medical records at Peking University First Hospital through a standardized process conducted by trained research staff. The collected variables included patient demographics (e.g., age, Body mass index [BMI]), ovarian reserve markers (e.g., AFC, AMH), treatment protocols (e.g., ovarian stimulation details, medication doses), and clinical outcomes (e.g., number of oocytes retrieved, embryo quality, and clinical pregnancy rate). To protect patient confidentiality, all data were anonymized and assigned unique identification codes. Systematic checks were implemented to ensure data accuracy and consistency, with any discrepancies resolved by reviewing the original records. Given the small number of missing cases, missing data were handled using complete case analysis without imputation [18].

### ART procedures

All study participants underwent controlled ovarian stimulation using Gonadotropin-releasing hormone (GnRH) antagonist protocols. Before starting ovarian stimulation, patients either received oral contraceptive pretreatment for 21 consecutive days or proceeded with IVF during natural menses. The decision to use OCs was made through a collaborative discussion between the clinical physicians and patients. The study prescribed three distinct formulations of OCs, comprising cyproterone acetate (2 mg)/ethinylestradiol (0.035 mg), desogestrel (0.15 mg)/ethinylestradiol (0.03 mg), and drospirenone (3 mg)/ethinylestradiol (0.03 mg). Controlled ovarian stimulation was initiated on Day 2 of the menstrual cycle with recombinant FSH and human menopausal gonadotrophin in a flexible dose based on age, body weight and ovarian reserve. Specifically, patients aged under 35 years and weighing ≤ 60 kg were started on 150–225 IU, while those aged over 35 years or weighing > 60 kg received 225–300 IU. GnRH antagonist was added when a leading follicle of 14 mm was achieved or when LH levels was above 5 IU/L. Oocyte maturation was induced by administering human chorionic gonadotrophin (hCG) when at least one follicle reached a diameter greater than 16 mm, and oocyte retrieval was performed 36 H after human chorionic gonadotrophin (hCG) administration. The oocytes were fertilized by either conventional IVF or by ICSI depending on sperm quality. Embryo development and quality were assessed at the cleavage stage based on ASEBIR embryo assessment criteria with minor modifications [19]. Fresh embryo transfer was generally performed on day 3 after oocyte retrieval with one to three embryos were transferred. The luteal phase was supported with progesterone gel, intramuscular progesterone and dydrogesterone the day of oocyte retrieval to 14 days after embryo transfer. Hormone treatment was continued for 8 weeks if pregnancy test was positive.

### Outcomes

The primary outcome of this study was the live birth rate, which was defined as the delivery of at least one viable fetus after 28 weeks of gestation. The secondary outcomes included the clinical pregnancy rate, which was defined as the detection of a gestational sac by transvaginal ultrasound 4 weeks after embryo transfer, and the incidence of a chemical pregnancy, which was characterized by a serum hCG level exceeding 10 IU/L 14 days after embryo transfer. In this study, available embryos were defined as those that had reached the 4-cell stage and exhibited ≤ 20% fragmentation by day 3 of development, while good-quality embryos were those that had attained the 7–10-cell stage with ≤ 20% fragmentation by day 3 of development. Pregnancy loss was defined as the occurrence of either spontaneous abortion or therapeutic abortion during gestation. Cycle cancellation was defined as the termination of IVF/ICSI cycles without oocyte retrieval or embryo transfer. The follicular output rate (FORT) was calculated as the ratio of preovulatory follicles on the hCG day (defined as follicles with a mean diameter of 16–22 mm) to the baseline AFC, multiplied by 100 [20]. The ovarian sensitivity index (OSI) was calculated as the number of oocytes collected after ovarian stimulation divided by the total dose of FSH administered multiplied by 1 [21].

### Statistical analysis

Statistical analyses were conducted using the Statistical Package for Social Sciences (SPSS) version 22.0 and R software version 4.2.2. Continuous variables were expressed as mean ± standard deviation and compared using either Student’s t-test or non-parametric Mann-Whitney U-test. Categorical variables were presented as frequency (percentage) and analyzed using either the chi-square test or Fisher’s exact test. To reduce the impact of confounding variables and identify comparable patients among different study groups, PSM was performed using the 1:3 nearest neighbor matching method with a caliper width of 0.01, implemented with the MatchIt package in R. Age, AFC, and AMH levels were assessed and compared between the groups after PSM. Important variables associated with clinical outcomes were analyzed using multivariable logistic regression and presented as odds ratios (OR) with 95% confidence intervals (CI). Statistical significance was set at a *P* value of less than 0.05.

## Results

A total of 2222 patients who met the POSEIDON criteria for POR were included in the study. As illustrated in Table 1, prior to matching, significant differences were observed between the two groups with respect to age, type of infertility, AMH, AFC and basal FSH. Patients in OCs group tended to be younger and exhibited better ovarian reserve. After a 1:3 nearest neighbor PSM, 369 patients in the OCs group were successfully matched to the 879 patients in the control group. No significant differences were observed between the two groups in terms of maternal age, BMI, duration of infertility, type of infertility and ovarian reserve markers. However, significant differences were found in the ovarian stimulation parameters between the two groups after PSM (Table 2). Patients in the OCs pretreatment group presented a better ovarian response to stimulation. Specifically, the level of estradiol on the day of hCG administration was higher in the OCs group (2250.24 ± 1094.11 versus 1878.22 ± 1199.37, *P* = 0.). The number of follicle ≥ 14 mm on the day of hCG administration (6.52 ± 3.67 versus 5.59 ± 3.29, *P* = 0.), as was the FORT (52.49 ± 21.85 versus 49.02 ± 23.72, *P* = 0.015), the number of oocytes retrieved (5.77 ± 2.25 versus 5.06 ± 2.50, *P* = 0.001), 2 pronuclei (PN) (3.52 ± 2.02 versus 3.00 ± 2.07, *P* = 0.), available embryos (3.75 ± 2.04 versus 3.21 ± 2.10, *P* = 0.) and good quality embryos (1.60 ± 1.45 versus 1.34 ± 1.39, *P* = 0.) were all significantly higher in the OCs group compared to the control group. Meanwhile, the OSI (2.40 ± 1.49 versus 1.97 ± 1.28, *P* = 0.) was significantly increased in patients who received OCs pretreatment. As for the clinical outcomes, the primary outcome of live birth rate (35.8% versus 30.0%, *P* = 0.047) was higher in the OCs pretreatment group, while the cancellation rate was lower in this group (Table 3). The biochemistry pregnancy rate, clinical pregnancy rate and pregnancy loss rate were similar between the two groups.

**Table 1:** Comparison of baseline characteristics between two treatment protocols before and after propensity score matching

Variable	Before matching			After matching		
	OCs (*n* = 401)	non-OCs (*n* = 1821)	*P* value	OCs (*n* = 369)	non-OCs (*n* = 879)	*P* value
Age (years)	32.35 ± 4.04	35.64 ± 5.09	0.^*^	33.54 ± 3.84	33.70 ± 3.75	0.414
BMI (kg/m^2^)	23.01 ± 3.47	22.73 ± 3.25	0.219	23.02 ± 3.47	22.62 ± 3.22	0.111
Infertility duration (years)	3.38 ± 2.59	3.71 ± 3.11	0.229	3.40 ± 2.65	3.63 ± 2.82	0.205
Type of infertility						
Primary n (%)	148(36.9)	863(47.4)	0.^*^	137(37.1)	348(39.1)	0.415
Secondary n (%)	253(63.1)	958(52.6)		232(62.9)	531(60.4)	
AMH (ng/ml)	1.91 ± 1.27	1.34 ± 1.13	0.^*^	1.84 ± 1.21	1.69 ± 1.20	0.690
AFC (n)	9.16 ± 4.05	7.24 ± 4.02	0.^*^	8.38 ± 3.73	8.20 ± 3.71	0.712
Basal E_2_ (pg/ml)	46.88 ± 22.57	46.49 ± 22.65	0.994	47.40 ± 22.72	46.00 ± 21.63	0.496
Basal FSH (IU/L)	8.73 ± 4.30	9.72 ± 4.48	0.^*^	8.89 ± 4.45	9.04 ± 3.47	0.531
Basal LH (IU/L)	4.63 ± 2.56	4.21 ± 2.56	0.107	4.58 ± 2.47	4.12 ± 2.14	0.162

All values presented as mean ± SD or n (%)

OCs, oral contraceptives; BMI, body mass index; AMH, anti-Müllerian hormone; AFC, antral follicle count; FSH, follicle-stimulating hormone; LH, luteinizing hormone; E_2_, estrogen

^*^ indicates statistically significant of *P*<0.05

**Table 2:** Comparison of ovarian stimulation parameters between two treatment protocols before and after propensity score matching

Variable	Before matching			After matching		
	OCs (*n* = 401)	non-OCs (*n* = 1821)	*P* value	OCs (*n* = 369)	non-OCs (*n* = 879)	*P* value
Total Gn dose (IU)	2695.92± 939.72	2674.20± 1082.84	0.927	2762.84± 931.77	2820.93± 1025.72	0.590
Duration of Gn (days)	9.66 ± 2.09	9.40 ± 2.53	0.053	9.64 ± 2.13	9.89 ± 2.30	0.077
E_2_ levels at the day of hCG (pg/ml)	2282.13± 1097.80	1618.97± 1173.90	0.^*^	2250.24± 1094.11	1878.22± 1199.37	0.^*^
LH levels at the day of hCG (IU/L)	2.33 ± 2.69	3.56 ± 4.91	0.^*^	2.42 ± 2.79	3.06 ± 3.61	0.^*^
P levels at the day of hCG (mg/ml)	0.90 ± 0.43	0.85 ± 0.47	0.017^*^	0.91 ± 0.43	0.90 ± 0.48	0.659
Follicles ≥ 14 mm at day of hCG (n)	6.76 ± 3.73	4.73 ± 3.32	0.^*^	6.52 ± 3.67	5.59 ± 3.29	0.^*^
Endometrial thickness (mm)	10.31 ± 2.38	10.42 ± 2.69	0.775	10.25 ± 2.37	10.85 ± 2.68	0.^*^
No. of oocytes retrieved (n)	5.85 ± 2.26	4.45 ± 2.69	0.^*^	5.77 ± 2.25	5.06 ± 2.50	0.001^*^
No. of 2PN (n)	3.64 ± 2.05	2.70 ± 2.13	0.^*^	3.52 ± 2.02	3.00 ± 2.07	0.^*^
Available embryos (n)	3.83 ± 2.85	2.04 ± 2.17	0.^*^	3.75 ± 2.04	3.21 ± 2.10	0.^*^
Good quality embryos (n)	1.65 ± 1.48	1.21 ± 1.39	0.^*^	1.60 ± 1.45	1.34 ± 1.39	0.^*^
OSI	2.53 ± 1.56	1.79 ± 1.31	0.^*^	2.40 ± 1.49	1.97 ± 1.28	0.^*^

All values presented as mean ± SD or n (%)

OCs, oral contraceptives; Gn, gonadotropin; E_2_, estrogen; LH, luteinizing hormone; P, progesterone; FORT, follicular output rate; PN, pronucleus; OSI, ovarian sensitivity index

^*^ indicates statistically significant of *P*<0.05

**Table 3:** Comparison of clinical outcomes between two treatment protocols before and after propensity score matching

Variable	Before matching			After matching		
	OCs (*n* = 401)	non-OCs (*n* = 1821)	*P* value	OCs (*n* = 369)	non-OCs (*n* = 879)	*P* value
Embryos transferred (n)	1.80 ± 0.69	1.54 ± 0.96	0.001^*^	1.78 ± 0.70	1.71 ± 0.82	0.133
Biochemistry pregnancy rate n (%)	192(47.9)	694(38.1)	0.^*^	173(46.9)	391(44.5)	0.437
Clinical pregnancy rate n (%)	169(42.1)	577(31.7)	0.^*^	153(41.5)	321(36.5)	0.100
Live birth rate n (%)	146(36.4)	465(25.5)	0.^*^	132(35.8)	264(30.0)	0.047^*^
Cycle Cancellation rate n (%)	27(6.7)	330(18.1)	0.^*^	27(7.3)	101(11.5)	0.027^*^
Pregnancy loss rate n (%)	16(4)	111(6.1)	0.100	14(3.8)	58(6.6)	0.053

All values presented as mean ± SD or n (%)

^*^ indicates statistically significant of *P*<0.05

To investigate the effectiveness of OCs pretreatment in specific groups of low prognosis patients, the POR patients were further categorized into four subgroups according to the POSEIDON criteria. Among these subgroups, no statistically significant differences were observed in terms of basic characteristic between those who underwent OCs pretreatment and the control group (supplementary Table 1).

The cycle characteristics presented Table 4 indicate that in group 1 patients, those who received OCs pretreatment required a lower dose of Gn (2478.64 ± 812.63 versus 2745.83 ± 963.13, *P* = 0.012) and a shorter duration of Gn treatment than those in non-OCs cycles. Furthermore, levels of estradiol on the day of hCG trigger (2438.16 ± 1128.60 versus 2179.83 ± 1191.82, *P* = 0.004), the FORT (46.95 ± 19.09 versus 42.38 ± 19.97, *P* = 0.021), the number of oocytes retrieved (6.49 ± 1.95 versus 5.94 ± 2.21, *P* = 0.035), 2PN (3.99 ± 1.93 versus 3.61 ± 2.12, *P* = 0.009), available embryos (4.20 ± 1.92 versus 3.76 ± 2.10, *P* = 0.031) and good quality embryos (1.73 ± 1.55 versus 1.52 ± 1.42, *P* = 0.031) were found to be significantly higher among POSEIDON group 1 patients who received OCs pretreatment. Additionally, the mean OSI (2.95 ± 1.45 versus 2.46 ± 1.40, *P* = 0.) was significantly higher in the OCs pretreatment group, indicating an increased ovarian response to stimulation in these patients. However, pretreatment with OCs did not significantly improve the ovarian response in POSEIDON group 2 to group 4 patients.

**Table 4:** Ovarian stimulation parameters of patients in the POSEISON subgroups

Variable	POSEIDON group1			POSEIDON group2			POSEIDON group3			POSEIDON group4		
	OCs (*n* = 189)	non-OCs (*n* = 414)	*P* value	OCs (*n* = 84)	non-OCs (*n* = 232)	*P* value	OCs (*n* = 58)	non-OCs (*n* = 128)	*P* value	OCs (*n* = 38)	non-OCs (*n* = 105)	*P* value
Gn dose (IU)	2478.64± 812.63	2745.83± 963.13	0.012^*^	3015.18± 768.79	3107.54± 961.87	0.591	3213.36± 1069.35	2662.20± 1173.51	0.483	2930.92± 1134.81	2675.71± 1098.71	0.475
Gn days (days)	9.87 ± 2.20	10.23 ± 2.10	0.028^*^	9.56 ± 1.67	10.17 ± 2.24	0.023^*^	9.41 ± 2.34	9.02 ± 2.64	0.548	9.05 ± 2.28	8.99 ± 2.28	0.900
E_2_ levels at the day of hCG (pg/ml)	2438.16± 1128.60	2179.83± 1191.82	0.004^*^	2403.68± 1022.60	2059.95± 1189.20	0.016^*^	1790.29± 904.50	1198.33± 937.58	0.^*^	1565.19± 880.46	1093.09± 811.43	0.014^*^
LH levels at the day of hCG (IU/L)	1.97 ± 1.50	2.49 ± 2.80	0.006^*^	2.36 ± 1.75	2.72 ± 1.88	0.045^*^	2.89 ± 2.94	4.07 ± 4.90	0.342	4.17 ± 6.62	4.80 ± 5.97	0.882
P levels at the day of hCG (mg/ml)	0.90 ± 0.45	0.94 ± 0.47	0.383	0.94 ± 0.47	0.91 ± 0.44	0.604	0.94 ± 0.35	0.81 ± 0.44	0.217	0.85 ± 0.36	0.84 ± 0.62	0.152
Follicles ≥ 14 mm at day of hCG (n)	7.72 ± 3.64	6.75 ± 3.22	0.023^*^	6.60 ± 3.44	5.92 ± 3.08	0.227	4.29 ± 2.58	3.24 ± 2.35	0.001^*^	3.61 ± 2.52	3.17 ± 2.08	0.530
Endometrial thickness (mm)	10.93 ± 2.33	11.30 ± 2.52	0.201	9.49 ± 2.09	10.84 ± 2.82	0.006^*^	9.86 ± 2.60	10.29 ± 2.59	0.998	9.08 ± 1.75	9.74 ± 2.67	0.087
No. of oocytes retrieved (n)	6.49 ± 1.95	5.94 ± 2.21	0.035^*^	5.77 ± 1.80	5.32 ± 2.29	0.100	4.62 ± 2.57	3.35 ± 2.24	0.064	3.95 ± 2.36	3.08 ± 2.21	0.081
No. of 2PN (n)	3.99 ± 1.93	3.61 ± 2.12	0.009^*^	3.58 ± 1.67	3.14 ± 1.96	0.068	3.02 ± 2.45	2.05 ± 1.66	0.097	2.21 ± 1.91	1.90 ± 1.75	0.997
Available embryos (n)	4.20 ± 1.92	3.76 ± 2.10	0.031^*^	3.79 ± 1.51	3.32 ± 2.02	0.057	3.28 ± 2.50	2.11 ± 1.69	0.113	2.13 ± 1.99	2.09 ± 1.77	0.417
Good quality embryos (n)	1.73 ± 1.55	1.52 ± 1.42	0.031^*^	1.55 ± 1.05	1.40 ± 1.44	0.380	1.59 ± 1.52	0.96 ± 1.10	0.050	1.11 ± 1.52	1.00 ± 1.38	0.989
OSI	2.95 ± 1.45	2.46 ± 1.40	0.^*^	2.09 ± 0.96	1.83 ± 0.98	0.098	1.72 ± 1.80	1.31 ± 0.91	0.722	1.39 ± 0.92	1.18 ± 0.79	0.921

All values presented as mean ± SD or n (%)

OCs, oral contraceptives; Gn, gonadotropin; LH, luteinizing hormone; E2, estrogen; P, progesterone; FORT, follicular output rate; PN, pronucleus; OSI, ovarian sensitivity index

^*^ indicates statistically significant of *P*<0.05

There was no difference in the mean number of embryos transferred for each patient between the four subgroups (Table 5). The clinical pregnancy rate (53.4% versus 42.5%, *P* = 0.012) and live birth rate (45.4% versus 36.2%, *P* = 0.030) were significantly higher in the OCs pretreatment group compared with the non-OCs group in POSEIDON group 1 patients. Moreover, the cancellation rate and pregnancy loss rate were similar between the two groups in POSEIDON group 1 patients. Similarly, the clinical pregnancy rates and live birth rates were higher in the POSEIDON group 3 patients, but the differences were not statistically significant.

**Table 5:** Clinical outcomes of patients in the POSEISON subgroups

Variable	POSEIDON group1			POSEIDON group2				POSEIDON group3			POSEIDON group4		
	OCs (*n* = 189)	non-OCs (*n* = 414)	*P* value	OCs (*n* = 84)	non-OCs (*n* = 232)	*P* value	OCs (*n* = 58)		non-OCs (*n* = 128)	*P* value	OCs (*n* = 38)	non-OCs (*n* = 105)	*P* value
Embryos transferred (n)	1.87 ± 0.52	1.81 ± 0.60	0.386	2.01 ± 0.65	1.92 ± 0.88	0.234	1.59 ± 0.77		1.30 ± 0.92	0.684	1.16 ± 1.00	1.35 ± 1.03	0.140
Biochemistry pregnancy rate n (%)	113(59.8)	216(52.2)	0.082	33(39.3)	106(45.7)	0.311	20(34.5)		38(29.7)	0.513	7(18.4)	31(29.5)	0.184
Clinical pregnancy rate n (%)	101(53.4)	176(42.5)	0.012^*^	28(33.3)	88(37.9)	0.454	18(31.0)		32(25.0)	0.390	6(15.8)	25(23.8)	0.304
Live birth rate n (%)	86(45.5)	150(36.2)	0.030^*^	25(29.8)	70(30.2)	0.944	18(31.0)		25(19.5)	0.085	3(7.9)	19(18.1)	0.135
Cancellation rate n (%)	6(3.2)	22(5.3)	0.247	3(3.6)	20(8.6)	0.127	7(12.1)		32(25.0)	0.045^*^	11(28.9)	27(25.7)	0.699
Pregnancy loss rate n (%)	10(5.3)	25(6.0)	0.716	2(2.4)	18(7.8)	0.083	2(3.4)		9(7)	0.507	2(5.3)	6(5.7)	1.000

All values presented as mean ± SD or n (%)

^*^ indicates statistically significant of *P*<0.05

A binary logistic regression analysis was conducted to analyze the effects of OCs pretreatment on clinical outcomes in all four subgroups of POSEIDON criteria. The analysis considered age, BMI, types of infertility, duration of infertility, basal FSH, AFC and AMH as confounding factors. The multivariate analysis revealed that OCs pretreatment had a positively correlation with live birth rate (adjusted OR 2.56, 95% CI 1.28–5.11, *P* = 0.008) and clinical pregnancy rate (adjusted OR 2.50, 95% CI 1.28–4.90, *P* = 0.008) in POSEIDON group 1 patients but did not show a significant relationship with pregnancy loss (Table 6). In contrast, no significant association was found between OCs pretreatment and any of these outcomes in POSEIDON group 2 to group 4 patients after controlling for important confounding factors (Supplementary Table 2).

**Table 6:** Logistic regression analysis for the effect of OCs pretreatment in POSEIDON group 1 patients

Variable	Unadjusted OR	95% CI	*P* value	Adjusted OR	95% CI	*P* value
Clinical pregnancy rate	1.55	1.10–2.19	0.013^*^	2.50	1.28–4.90	0.008^*^
Live birth rate	1.47	1.04–2.08	0.031^*^	2.56	1.28–5.11	0.008^*^
Pregnancy loss rate	0.87	0.41–1.85	0.716	0.65	0.14–3.02	0.586

OR, Odds ratio; CI, Confidence interval. Maternal age, BMI, AFC, duration of infertility, type of infertility, AMH and basal FSH were adjusted in the multivariate analysis

^*^ indicates statistically significant of *P*<0.05

## Discussion

In the present study, we observed that pretreatment with OCs may enhance the ovarian response to exogeneous gonadotropins and improve the clinical outcomes in IVF/ICSI in POSEIDON group 1 patients. However, we did not find a similar beneficial effects of OCs pretreatment in low prognosis patients in POSEIDON group 2 to group 4.

Oral contraceptives are a widely adopted pretreatment strategy for scheduling in IVF/ICSI cycles, as well as synchronization of follicular development in ART worldwide [8, 9]. During the early stages of the follicular phase in the menstrual cycle, small antral follicles display divergent sizes, ranging from 2 to 9 mm in diameter, and heterogeneous antral follicles exhibit varying degrees of sensitivity to FSH, which may lead to a slight decrease in oocyte yield and reduced pregnancy rates during ovarian stimulation [22, 23]. The precise mechanism underlying the differences in antral follicle size is still uncertain. One possible explanation for this phenomenon is that some small antral follicles in the luteal phase are able to respond to lower levels of FSH and initiate early development [24]. In patients with diminished ovarian reserve or advanced maternal age, elevated FSH levels may accelerate the development of sensitive follicles, thereby amplifying size discrepancies during the follicular phase [25]. Hence, it is essential to minimize the physiologic heterogeneity of small antral follicles, and the use of OCs is considered a useful option.

Before ovarian stimulation, OCs are administered to suppress endogenous gonadotropin secretion via negative feedback, thereby preventing spontaneous production of LH and FSH, and ultimately increasing the number of oocytes retrieved and clinical pregnancy rates [13, 26]. In the present study, our findings revealed that pretreatment with OCs resulted in a significant reduction in the total amount of gonadotropins required to achieve ovarian response, as well as a shorter duration of ovarian stimulation in POSEIDON group 1 patients. To further assess follicular synchronization, we evaluated the FORT, which measures the proportion of retrieved follicles relative to the available antral follicle count. In POSEIDON group 1, the FORT value was notably higher in the OCs group, reflecting enhanced synchronization of follicular development and improved ovarian responsiveness. These findings underscore the critical role of OCs pretreatment in minimizing follicular heterogeneity and optimizing ovarian stimulation outcomes, particularly in young, unexpected low-prognosis patients. Additionally, OCs pretreatment was associated with a significant increase in the number of oocytes retrieved, 2PN, available embryos and good quality embryos. Reduced gonadotropin consumption and higher available embryos during ovarian stimulation after OCs pretreatment were also reported by Lu et al. [16]. Likewise, Huirne et al. found that the number of oocytes retrieved (13.5 ± 6.7 versus 10.2 ± 6.0, *P* < 0.01) and number of good quality embryos (5.3 ± 4.5 versus 3.7 ± 4.1, *P* < 0.01) were higher in the OCs pretreatment group [27]. Besides, a case series examining patients with premature ovarian insufficiency revealed a total number of 48 oocytes retrieved in 17 patients, although the study lacked a control group to prove the efficacy of OCs pretreatment [10].

However, intriguingly, OCs pretreatment did not yield an improvement in the ovarian response for patients within POSEIDON group 2 to group 4, which is in line with a previous study conducted in POR patients [13]. Nevertheless, it’s worth noting that the inclusion criteria in the previous study were irrespective of age and AMH stratification, which could conceal the meaningful differences in pretreatment effects on the ovarian response [13]. Moreover, the specific benefit of OCs pretreatment in POSEIDON group 1 patients may be explained by their characteristic unexpected poor ovarian response to ovarian stimulation, which can be caused by resistance to exogenous gonadotropins and may be related to single nucleotide polymorphism in FSH receptor [28]. Pretreatment with OCs may increase the sensitivity of FSH receptor to exogenous gonadotropin and lead to improved ovarian response in these patients. In addition, concerning POSEIDON group 2 and 4, patients aged over 35 years tend to exhibit relatively lower pregnancy rate and live birth rate compared with their younger counterparts. Some researchers have posited that estradiol levels are lower in patients over 35 years old than in the younger age group, indicating a greater degree of pituitary suppression in older patients [29, 30]. Therefore, it is reasonable to hypothesize that the recovery from gonadotrophin suppression, which occurs after OCs pretreatment, may outweigh the benefits of follicular synchronization in older patients, potentially explaining the result in this study.

Although pretreatment with OCs may aid in synchronizing follicle development during ovarian stimulation, several studies have suggested that using OCs as pretreatment for patients with regular menses or PCOS may have a detrimental effect on outcomes following fresh embryo transfer [16, 31]. In a recent meta-analysis comprising six randomized controlled studies, Farquhar et al. indicated that OCs pretreatment was associated with a lower ongoing pregnancy rate and live birth rate in the antagonist protocol after fresh embryo transfer (OR 0.74, 95% CI 0.58–0.95) [8]. Similarly, some studies have reported that OCs pretreatment had an adverse effect on clinical outcomes during the GnRH agonist and antagonist cycles in PCOS patients [15, 32, 33]. In accordance with these studies, Lu et al. reported that both live birth rate (42.6% versus 52.8%) and cumulative live birth rate (62.8% versus 67.6%) were decreased in normal ovulatory patients who received OCs pretreatment [16]. One possible explanation for the negative effect on the clinical outcomes in fresh cycles is that the diminished endometrial thickness in OCs pretreatment resulted in a lower birth rate in fresh cycle and the lack of impact on cumulative live birth rate further demonstrated the suspicion. Besides, some authors believe that OCs pretreatment may induce advanced endometrium maturation, resulting in asynchrony between the endometrium and embryos [34]. However, conflicting results have been reported regarding the impact of OCs pretreatment on IVF outcomes. In contrast to these studies, a prospected randomized study performed by Kim et al. showed that OCs pretreatment in the GnRH antagonist protocol could help to increase the clinical pregnancy rate (33.3% versus 22.2%) and live birth rate (29.6% versus 18.5%) in POR patients, although significant differences were not found among the groups [13]. Similarly, Lindheim et al. reported that short-term gonadotropin suppression with OCs significantly increased pregnancy rates in POR patients [34]. The long period of using OCs in PCOS patients before ovarian stimulation may result in the differences in endometrium thickness and IVF outcomes compared to the POR patients.

Our data are consistent with previous studies in POR patients and indicate that the clinical pregnancy rate and live birth rate were significantly higher after OCs pretreatment in POSEIDON group 1 patients, with no differences in endometrial thickness observed in the treatment groups [13]. Although the cancellation rate of cycles and pregnancy loss rate were lower with OCs pretreatment, these differences failed to reach statistical significance. In POSEIDON group 3 patients, OCs pretreatment slightly improved ovarian response and clinical outcomes, but the sample size was insufficient to achieve significance. No significant benefits of OCs pretreatment were observed in patients in group 2 and group 4. Overall, our data suggest that OCs pretreatment improves the ovarian response and clinical outcomes in young unexpected low prognosis patients.

The main strength in this study is its novelty in demonstrating the impact of OCs pretreatment on clinical IVF outcomes among various low prognosis patients based on the POSEIDON criteria, which consider the broad heterogeneity in patients with POR. Nonetheless, there are several limitations to this study. Firstly, patients were not randomly assigned to receive OCs or to undergo natural menstruation, resulting in imbalanced baseline characteristics. Although PSM and logistic regression were employed to minimize confounding factors, some potential confounders may have been overlooked, and selection bias may still exist. Secondly, three different types of OCs with varying progesterone components were employed, and it remains uncertain whether the outcomes differ based on the specific type of OC used. Additionally, the sample size for POSEIDON groups 3 and 4 was relatively small, making it difficult to detect significant differences in clinical outcomes. Moreover, cumulative live birth rate might serve as a more appropriate outcome for evaluating the efficacy of OCs as pretreatment in low prognosis patients. Thus, a well-designed, multicenter, prospective randomized controlled trial is necessary to further validate the results.

## Conclusion

In conclusion, our findings indicate that pretreatment with oral contraceptives is a more favorable option compared to waiting for spontaneous menses in POSEIDON group 1 low prognosis patients prior to ovarian stimulation using GnRH antagonist protocol for IVF. Young patients with suboptimal ovarian response had improved live birth rate and ovarian response when using OCs, compared to those who did not use OCs. However, this benefit was not observed in POSEIDON group 2 to group 4. It is important to weigh the advantages and disadvantages of OCs pretreatment in different subgroups of patients with POSEIDON criteria when making clinical decisions.

## Electronic supplementary material

Below is the link to the electronic supplementary material.

Supplementary Material 1

Supplementary Material 2

## Data Availability

No datasets were generated or analysed during the current study.

## Abbreviations

POR: Poor ovarian response
OCs: Oral contraceptives
POSEIDON: Patient-oriented strategies encompassing individualized oocyte number
ART: Assisted reproduction technologies
Gn: Gonadotropins
ESHRE: European society of human reproduction and embryology
AMH: Anti-Mullerian hormone
AFC: Antral follicle count
LH: Luteinizing hormone
PCOS: Polycystic ovarian syndrome
IVF: In-vitro fertilization
ICSI: Intracytoplasmic sperm injection
FSH: Follicle stimulating hormone
PSM: Propensity score matching
GnRH: Gonadotropin-releasing hormone
BMI: Body mass index
hCG: human chorionic gonadotrophin
OSI: Ovarian sensitivity index
SPSS: Statistical package for social sciences
OR: Odds ratios
CI: Confidence intervals
