# Supplementary material for: Pretreatment with oral contraceptives benefit POSEIDON group 1 low prognosis patients during GnRH-antagonist protocol: a propensity score-matched retrospective cohort study
Source: J Ovarian Res. 2025 Mar 7;18:47. doi: 10.1186/s13048-025-01613-6 (PMC11889746; doi:10.1186/s13048-025-01613-6)
Supplement: Supplementary file 1 — Supplementary Material 1 [file 13048_2025_1613_MOESM1_ESM.docx]

**Supplementary Table 1** Baseline characteristics of patients in the POSEIDON subgroups.

| Variable | POSEIDON group1 | | | POSEIDON group2 | | | POSEIDON group3 | | | | POSEIDON group4 | | |
| --- | --- | --- | --- | --- | --- | --- | --- | --- | --- | --- | --- | --- | --- |
|  | OCs  (n=189) | non-OCs  (n=414) | P  value | OCs  (n=84) | non-OCs  (n=232) | P  value | | OCs  (n=58) | non-OCs  (n=128) | P  value | OCs  (n=38) | non-OCs  (n=105) | P  value |
| Age (years) | 30.47±2.68 | 31.02±2.48 | 0.089 | 37.10±1.90 | 36.99±1.64 | 0.821 | | 31.41±2.32 | 31.17±2.65 | 0.891 | 36.87±1.88 | 37.20±2.00 | 0.485 |
| BMI (kg/m^2^) | 23.03±3.48 | 22.45±3.09 | 0.158 | 23.29±3.69 | 22.84±3.25 | 0.460 | | 22.72±3.48 | 22.48±3.27 | 0.961 | 22.79±2.98 | 22.98±3.60 | 0.849 |
| Infertility duration (years) | 3.20±2.07 | 3.20±2.30 | 0.987 | 4.11±3.70 | 4.54±3.49 | 0.149 | | 2.99±2.48 | 2.98±1.91 | 0.750 | 3.46±2.53 | 4.11±3.36 | 0.946 |
| Type of infertility |  |  |  |  |  |  | |  |  |  |  |  |  |
| Primary n (%) | 133(70.4) | 274(66.2) | 0.309 | 39(46.4) | 128(55.2) | 0.169 | | 42(72.4) | 82(64.1) | 0.263 | 20(52.6) | 58(55.2) | 0.782 |
| Secondary n (%) | 56(29.6) | 140(33.8) |  | 45(53.6) | 104(44.8) |  | | 16(27.6) | 46(35.9) |  | 18(47.4) | 47(44.8) |  |
| AMH (ng/ml) | 2.63±1.09 | 2.66±1.03 | 0.774 | 2.31±1.07 | 2.34±0.90 | 0.196 | | 0.81±0.34 | 0.70±0.45 | 0.106 | 0.83±0.51 | 0.74±0.44 | 0.361 |
| AFC (n) | 10.52±2.71 | 9.97±3.45 | 0.050 | 9.04±3.21 | 8.42±3.16 | 0.342 | | 4.81±1.66 | 4.83±1.90 | 0.076 | 3.89±1.01 | 4.17±1.85 | 0.562 |
| Basal E_2_ (pg/ml) | 46.16±20.54 | 44.53±19.12 | 0.392 | 49.89±24.03 | 46.10±20.59 | 0.866 | | 46.74±21.02 | 47.81±27.33 | 0.564 | 49.40±32.29 | 49.88±25.65 | 0.231 |
| Basal FSH (IU/L) | 7.51±2.36 | 7.91±1.96 | 0.151 | 8.74±3.94 | 9.01±2.53 | 0.417 | | 11.35±5.76 | 11.31±5.66 | 1.000 | 12.87±7.23 | 11.06±4.49 | 0.148 |
| Basal LH (IU/L) | 4.67±2.53 | 4.06±1.70 | 0.301 | 4.33±2.42 | 4.11±2.15 | 0.893 | | 4.56±2.10 | 4.33±3.24 | 0.114 | 4.68±2.80 | 4.16±2.20 | 0.836 |

All values presented as mean ± SD or n (%).

OCs, oral contraceptives; BMI, body mass index; AMH, anti-Müllerian hormone; AFC, antral follicle count; FSH, follicle-stimulating hormone; LH, luteinizing hormone; E_2_, estrogen

^*^ indicates statistically significant of P＜0.05
