# Supplementary material for: Pretreatment with oral contraceptives benefit POSEIDON group 1 low prognosis patients during GnRH-antagonist protocol: a propensity score-matched retrospective cohort study
Source: J Ovarian Res. 2025 Mar 7;18:47. doi: 10.1186/s13048-025-01613-6 (PMC11889746; doi:10.1186/s13048-025-01613-6)
Supplement: Supplementary file 2 — Supplementary Material 2 [file 13048_2025_1613_MOESM2_ESM.docx]

**Supplementary Table 2** Logistic regression analysis for the effect of OCs pretreatment on rates of

clinical pregnancy, live birth and pregnancy loss in POSEIDON group 2 to group 4 patients

| Variable | Unadjusted OR | 95% CI | P value | Adjusted OR | 95% CI | P value |
| --- | --- | --- | --- | --- | --- | --- |
| Group 2 |  |  |  |  |  |  |
| Clinical pregnancy rate | 0.82 | 0.48-1.38 | 0.454 | 0.79 | 0.35-1.80 | 0.574 |
| Live birth rate | 0.98 | 0.57-1.69 | 0.944 | 0.72 | 0.32-1.65 | 0.436 |
| Pregnancy loss rate | 0.29 | 0.07-1.28 | 0.102 | 1.26 | 0.70-2.28 | 0.438 |
| Group 3 |  |  |  |  |  |  |
| Clinical pregnancy rate | 1.35 | 0.68-2.68 | 0.391 | 1.55 | 0.66-3.63 | 0.309 |
| Live birth rate | 1.85 | 0.91-3.76 | 0.087 | 1.80 | 0.76-4.26 | 0.181 |
| Pregnancy loss rate | 0.47 | 0.10-2.26 | 0.347 | 0.93 | 0.15-5.84 | 0.937 |
| Group 4 |  |  |  |  |  |  |
| Clinical pregnancy rate | 1.35 | 0.68-2.68 | 0.391 | 1.55 | 0.66-3.63 | 0.309 |
| Live birth rate | 1.85 | 0.91-3.76 | 0.087 | 1.80 | 0.76-4.26 | 0.181 |
| Pregnancy loss rate | 0.47 | 0.10-2.26 | 0.347 | 0.93 | 0.15-5.84 | 0.937 |

OR, Odds ratio; CI, Confidence interval. Maternal age, BMI, AFC, duration of infertility, type of infertility, AMH and basal FSH were adjusted in the multivariate analysis.
